# Direct observation of the specific heat of Majorana quasiparticles in superfluid^3^He-B

**DOI:** 10.1038/s41598-020-77128-5

**Published:** 2020-11-18

**Authors:** Yu. M. Bunkov, R. R. Gazizulin

**Affiliations:** 1 Russian Quantum Center, Skolkovo, Moscow, Russia 143025; 2 Univ. Grenoble Alpes, Inst. NEEL-CNRS, 38042 Grenoble, France

**Keywords:** Physics, Condensed-matter physics, Quantum physics

## Abstract

The existence of Majorana quasiparticles was predicted in the edge state in topological insulators, especially in the p-wave superfluid medium ${^3}$He-B. Due to its purity and coherent quantum state, ${^3}$He-B is an ideal platform for searching for Majorana fermions in condensed matter systems. In the limit of extremely low temperatures, the density of Bogolyubov quasiparticles and the heat capacity of ${^3}$He-B decrease exponentially. In this article, we present the first observation of the deviation of its heat capacity from exponential dependence in the limit of record low cooling. We found an additional heat capacity that more than doubled the heat capacity of bulk ${^3}$He-B and changes as T$^2$. The additional heat capacity is in good agreement with the predicted heat capacity of 2D gas of Majorana. This observation is a direct proof of the existence of Majorana quasiparticles in ${^3}$He-B.

## Majorana fermions in superfluid ${^3}$He-B

Majorana’s fermion was predicted in 1937 by Majorana^1^ as a particles that are identical to its own antiparticles. Although no one has discovered an elementary particle corresponding to the Majorana fermion, there are quasiparticles in some special classes of condensed matter systems that must have Majorana properties^2–4^. In particular, these quasiparticles can exist at the edge of superfluid ${^3}$He-B^5^.

**Figure 1:**
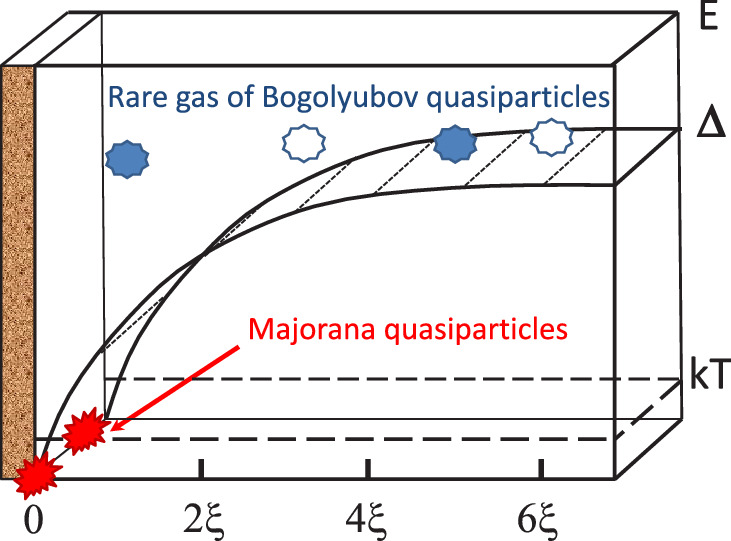
The dependence of the energy gap $\Delta$ in superfluid ${^3}$He-B on the distance from the sample edge in units of the coherence length is shown. This gap is in 20 times bigger then *kT* at the conditions of experiments and Bogolyubov QPs gas is very diluted. Majorana QPs are located near the surface at a distance of the coherence length. They can move along the surface due to nonzero kinetic energy, and contribute to the heat capacity proportional to the square of the temperature.

At the critical temperature $T_{c}$, which varies between 0.93 and 2.49 mK depending on the pressure, ${^3}$He goes through 2nd order phase transition into its superfluid states, those are unique in their richness. The key factor of superfluidity is the formation of Cooper pairs, ${i.e}$ pairs of ${^3}$He, which form the ground state of the system. The dispersion relation for elementary excitations, ${ i.e}$ broken Cooper pairs (the Bogolyubov quasiparticles (QPs)), above this state is:

1 $$\begin{aligned} E=\sqrt{\eta ^{2}+\Delta ^{2}}, \end{aligned}$$

where $\Delta$ is the temperature-dependent gap parameter and $\eta$ is the kinetic energy of the excitation. A distinctive feature of this dispersion relation is the absence of excitations with $E<\Delta$. However, in the region near the edge of the cell, the energy gap drops to zero at a distance corresponding to the superfluid coherence length $\xi$ (Fig. 1). In this region excitations can exist in the form of a two-dimensional gas of quasiparticles with Majorana properties, as was predicted in^6,7^. This follows from the symmetry of the particle-hole of the Bogolyubov QPs ($\gamma$); $\gamma _{E}^{+}=\gamma _{E}^-$. At zero energy $\gamma _{0}^{+}=\gamma _{0}^-$, which tells us that quasiparticles with zero energy are the same as quasi-holes, and therefore has a properties of Majorana.

The Majorana QPs in ${^3}$He-B can be detected by anomalous attenuation of transverse sound^8,9^ and by mobility of negative ions. These investigations are presented in detail in the contemporary review^10^. The topic review of theoretical and experimental methods of Majoraana investigations one can found in^11^. A direct proof of the existence of Majorana quasi particles can be the separation of their heat capacity from the heat capacity of superfluid ${^3}$He-B. The point is that the heat capacity of ${^3}$He-B decreases exponentially with decreasing temperature, while the heat capacity of the Majorana QPs decreases only as the square of the temperature. The method of separating two different dependences of heat capacity on temperature is used in this article.

The experiments were carried out on a powerful ultra-low-temperature facility in the Institute Neel in Grenoble^12^. This facility achieved a world record for condensed matter cooling of 0.1 mK. We used this setup to develop a superfluid ${^3}$He-B based dark matter detector^13–15^. Detailed studies of the thermometry and heat capacity of ${^3}$He-B^16–18^, as well as the energy of interaction with neutrons, muons^19^ and $\beta$ radiation^20^ have been accurately investigated. In particular, a method for discriminating electronic and nuclear events was developed, based on a different time constant of temperature recovery after events^21^.

Calibration of the thermal properties of superfluid $^3$He made it possible to carry out experiments with a fast second-order transition for quantum matter. We heated a small region of superfluid $^3$He using a fusion reaction of a neutron with a nucleus $^3$He. Then the superheated region rapidly cooled in analogy with the cooling of the universe after the Big Bang. It turned out that part of the thermal energy is not converted into the energy of quasiparticles. The missing energy was explained by the creation of quantum vortices in accordance with the Kibble-Zurek theory, developed for the formation of cosmological strings in the universe after the Big Bang^22^. A quantitative agreement with the theory has been achieved.

We found that at temperatures below 0.15 mK, the detector’s sensitivity drops sharply and deviates from theoretical values. First, we linked this deviation to thermometry and calibration heat pulses at such low temperatures^19^. However, later we noticed that the cooling time constant after the fusion reaction with the neutron also rises sharply, which cannot be explained by problems with thermometry and calibration heat pulses^23^.

It became clear that the heat capacity of ${^3}$He in the experimental cell deviates from the theoretical curve. One of the possible explanations could be associated with a layer of adsorbed ${^3}$He on the cell walls. We have thoroughly studied the heat capacity of the adsorbed solid ${^3}$He^24^ and found that its heat capacity changes as a $T^{-2}$ with cooling. Since the layer of adsorbed ${^3}$He greatly reduces the sensitivity of the detector, a technique was developed to exclude its appearance in the cell. We filled the experimental cell at a relatively high temperature with ${^4}$He gas, so that upon cooling, it had to cover all surfaces with excess layers. Then we flooded the chamber with liquid ${^3}$He at a temperature of about 10 mK. Since the adsorption energy of ${^3}$He is much lower, it cannot tear ${^4}$He off the surface. Thus, the presence of adsorbed solid ${^3}$He was practically excluded.

The most probable source for the appearance of additional heat capacity is the presence of Majorana quasiparticles on the cell surface. To confirm this assumption, we performed experiments with cells with different volume-to-surface ratios, the results of which we present in this article. Preliminary results with only one cell have been posted in^25^.

## Majorana and Bogolubov QPs heat capasity

A direct consequence of the Bogolyubov QPs energy gap is the exponential decreasing of $^3$He-B heat capacity with cooling:

2 $$\begin{aligned} C_{bulk} \sim V \, k_F^2\, \xi ^{-1} \left( \frac{\Delta }{kT}\right) ^{3/2} \left( 1+\frac{21kT}{16\Delta }\right) \exp \;\left( - \frac{\Delta }{kT}\right) \end{aligned}$$

where $\xi$ is the $^3$He-B coherence length, $k_F$ is the Fermi momentum, $\Delta$ is the superfluid gap $\simeq 2kT_c$ and *V* is the volume of the sample.

The question about the heat capacity of Majorana quasiparticles on the surface is more complicated. A similar problem was investigated for electrons in cuprate superconductors^26^. It was shown that the heat capacity depends on the density of impurities and may be written as^27^

3 $$\begin{aligned} C_{maj} \sim S \, k_F^2\, \left( \frac{\Delta }{kT}\right) ^{-(1 + Z)}, \end{aligned}$$

Here *S* is the surface area of the cell. *Z* is a parameter, related to a roughness of the surface on a characteristic length about few $\xi$. Parameter $Z = 1$ for smooth surface and decreases to zero in the case of roughness.

The ratio of these heat capacities, including the numerical factors, reads:

4 $$\begin{aligned} \frac{C_{maj}}{C_{bulk}} = \frac{\pi ^3}{8\sqrt{2}} \frac{ \xi S}{V} \, \left( \frac{\Delta }{kT}\right) ^{-(5/2 +Z)}\exp \, \left( \frac{\Delta }{kT}\right) . \end{aligned}$$

From Eq. (3) it can be seen that depending on surface-to-volume ratio Majorana QPs may have significant contribution to the ${^3}$He heat capacity (in comparison with the heat capacity of Bogolyubov QPs) below some low enough temperature. We have measured the heat capacity of $^3$He-B samples in two cells with very different volume-to-surface ratios at zero pressure as a function of temperature down to about 0.1 mK.

## Precise heat capacity measurements

To measure the heat capacity of superfluid ${^3}$He, we used the prototype dark matter detector described in^16^. It consists of a cooper box filled with ${^3}$He. The box has a tiny hole on one side that is used as a thermal connection to the surrounding ${^3}$He. Due to the Kapitza resistance, ${^3}$He is thermally separated from the cell walls, and thermalization occurs only through the hole. Two vibrating wire resonators (VWRs) were installed inside the bolometer to release some calibrated amount of energy and measure the corresponding temperature response. The VWR consists of an extremely thin (4 $\mu m$) superconducting NbTi wire, fixed at both ends in a plane, and has an approximately semicircular shape. Instantaneous measurements of the VWR amplitude under constant excitation give the time dependence of the QP density in the superfluid ${^3}$He^28^. Studies of VWR dumping have shown that the density of quasiparticles decreases exponentially with decreasing temperature and, accordingly, the heat capacity also changes exponentially. Accordingly, the half-width of VWR *W*(*T*) follows to equation:

5 $$\begin{aligned} W(T)\sim \alpha \exp (-\Delta /kT), \end{aligned}$$

where $\alpha \sim 10^5$ Hz is pre-factor which depends on geometry of the wire and pressure. It was calibrated against Pt NMR thermometer in Lancaster^29^ and Grenoble^18^. The computer programs, which calculate parameter $\alpha$ for different parameters of the VWR are available in Grenoble and Lancaster.

One of the VWRs was used to inject a calibrated amount of energy inside the bolometer. This method of heating ${^3}$He, proposed in^13^, is based on the scattering of a wire with existing QPs (at a low speed) or even exciting new QPs at a speed of wire approaching the critical one. In the second case, the process of heating $^3$He is very efficient. By driving the VWR with a current at its resonant frequency for a short time, a controlled amount of energy can be injected into the system. It can be calculated by integrating the electrical power $E_{electric} = \int UIdt$. The electrical energy is first converted into kinetic/potential energy of the heating wire and then dissipated into the fluid through a speed-dependent frictional coupling.

After reaching an operating temperature of about 100 $\mu$K, the first VWR switches to monitoring mode, which allows us to determine the temperature with a time resolution of less than 1 s. Then a heating pulse of approximately 70 ms is applied to the second VWR to inject an energy of 100 - 1000 keV into the bolometer. After the heating pulse, the temperature inside the cell rises and then returns to the initial temperature due to thermalization through the hole with a certain time constant. This temperature response is recorded by the first VWR as an increase in its resonance width and, accordingly, a decrease in the VWR oscillation amplitude.

**Figure 2:**
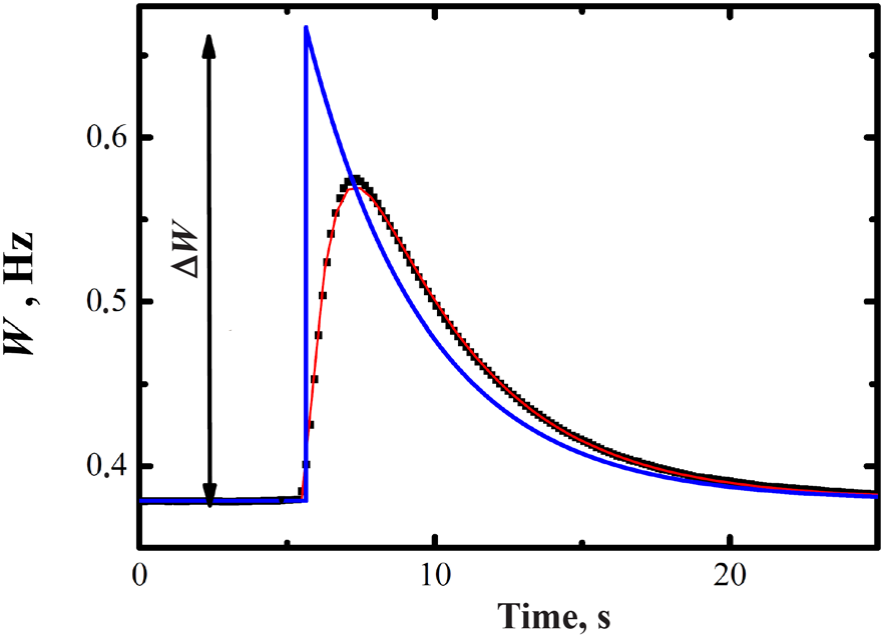
The evolution of the VWR width after a heating pulse. The points are the experimental data, the line—its fit by Eq. (6). The second line (blue in color) is the VWR width calculated in accordance with Eq. (6) for the case if $\tau _{R} =0$. This line should be proportional to the instantaneous density of quasiparticle.

After the heating pulse, the density of quasiparticles and the temperature increase almost instantaneously. The thermometry VWR amplitude decreases with a certain time delay due to the VWR Q factor. The response time $\tau _{R}$ is inversely proportional to the baseline width of the VWR ($W_{0}$); $\tau _{R}=1/\left( \pi W_{0} \right)$. After an energy deposition the quasiparticles density rises rapidly and then decreases with a time scale, $\tau _b$, of a few seconds, as shown in Fig. 2. The $\tau _b$ is the time scale of flow out of quasiparticles from the bolometer. Consequently, the time dependence of measured VWR response on the heating pulse at $t_{0}$ can be written:

6 $$\begin{aligned} W\left( t \right) =W_{0}+\Delta W\frac{\tau _{b}}{\tau _{b}-\tau _{R}}\left[ \left( \exp \left( -\frac{t-t_{0}}{\tau _{b}} \right. \right) -\exp \left( -\frac{t-t_{0}}{\tau _{R}} \right) \right] \end{aligned}$$

where $\Delta W$ is the fitting parameter, the broadening of VWR immediately after the heat pulse, for the conditions of zero time delay of VWR thermometer. We have fitted our data using this function as shown in Fig. 2 and tabulated the values of $\Delta W$ as function of the temperature and heating pulse.

To relate the increase in width VWR $\Delta W$ after a heat pulse with the corresponding heat release *U*, we define the calibration coefficient $\sigma$ as

7 $$\begin{aligned} \sigma =\frac{\Delta W}{U}=\frac{1}{C(T)}\frac{dW(T)}{dT}, \end{aligned}$$

where *C*(*T*) is the heat capacity of ${^3}$He. *W*(*T*) is obtained by the Eq. (5). By calibrating the dark matter detector, we found that the calibration coefficient $\sigma$ increases with cooling, which is in good agreement with an exponential decrease in heat capacity to temperatures on the order of 0.15 $\hbox {T}_c$. However, below this temperature, the detector’s sensitivity decreases sharply. The original explanation put forward in the work^19^ contradicts the data on the recovery of the detector temperature after the reaction with the neutron^23^, which can only be explained by the real deviation of the heat capacity from exponential decrease. It can be assumed that the deviation of the heat capacity is related to the contribution to the heat capacity of Majorana quasiparticles, which exists on the chamber surface. Therefore, we carried out a systematic study of the heat capacity in two chambers with different volume-to-surface ratios.

We used two different cells. The first is the cell used for the dark matter detector. The heat capacity of ${^3}$He-B in this cell has been well studied over a wide temperature range^16,31^. The second cell was made from the same material as the first, but slightly larger. In order to increase the surface inside the cell, a set of copper foils was inserted into it. As a result, the surface-to-volume ratio was increased 20 times. We can estimate the geometric surface of the first cell (cell *A*) $S \sim$ 113 mm${^ 2}$ and volume $V \sim$ 130 mm${^ 3}$, and in the second (cell *B* ) $S \sim$ 3216 mm${^ 2}$ and $V \sim$ 171 mm${^ 3}$. Another important difference between the cells concerns the VWR wire diameters. In cell *A* we used VWR made of 4 $\mu$m wire, and in cell *B* 10 $\mu$m. It is known that the diameter of VWR wires determines the temperature range in which they have the best sensitivity. The lowest temperature that can be measured by VWR is associated with reduced wire diameter, but thicker wires are more sensitive at higher temperatures. After eq. (4) it should be expected that ${a}$ ${priori}$, that in the cell *B* the heat capacity of the Majorana QPs can have a significant contribution at higher temperatures than in the cell *A*. Therefore, we installed a VWR from a wire of a larger diameter into the *B* cell.

## The experimental results

**Figure 3:**
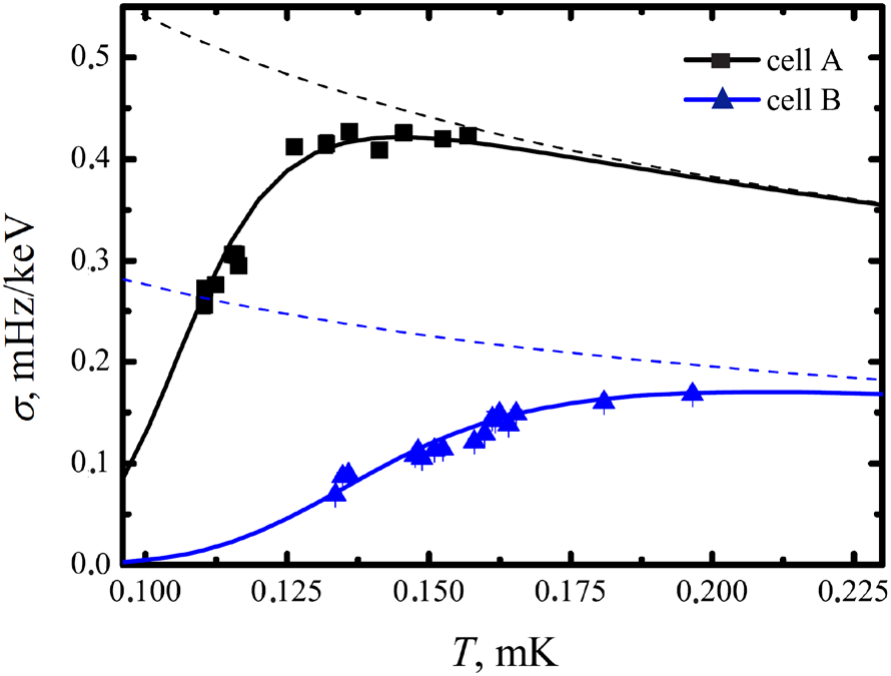
Experimental results of measurements of the calibration factor for VWR in two cells with different surface-to-volume ratio at 0 bar. The black dashed line shows the previous calibration of the dark matter detector (cell A) at temperatures above 0.15 $\hbox {T}_C$. The blue dashed line corresponds to the calibration for cell B. It was calculated from the black one taking into account the difference in cell volume and VWR wire diameters. Solid lines correspond to the adjustment of the additional heat capacity due to the Majorana QPs.

In this article, we present a systematic study of the calibration coefficient $\sigma$ for two cells with different surface to volume ratios. The temperature dependence of $\sigma$ for both cells at 0 bar is shown in Fig. 3. All necessary corrections, such as the response time of the VWR and the temperature dependence of the thermalization time after the heating pulse, are already included. This procedure has been well developed for the dark matter detector^16,17,31^. The black dashed line shows the previous calibration of the dark matter detector (cell A) at temperatures above 0.15 $\hbox {T}_C$. It corresponds well to the heat capacity of the Bogolyubov QPs gas. The blue dashed line corresponds to the calibration for cell B. It was calculated from the black one taking into account the difference in cell volume and VWR wire diameters.

**Figure 4:**
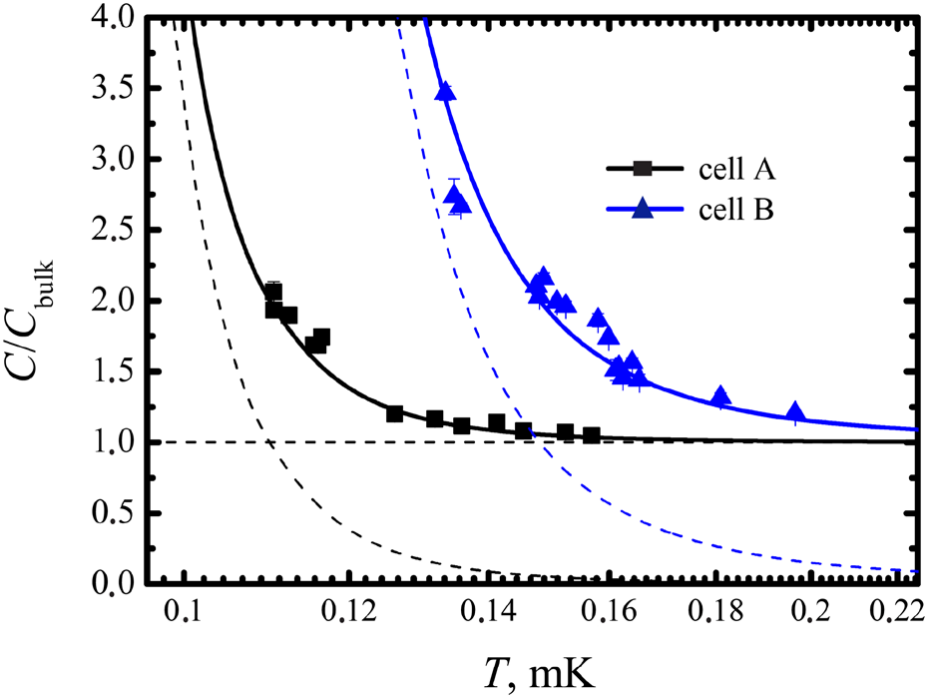
Shown are the experimental points for the heat capacity in two cells, normalized to the heat capacity calibration at high temperatures ($C/C_{bulk}$). The additional heat capacity is fitted by a power-law dependence on temperature and is shown by dashed lines, black for the cell *A* and blue for the cell *B*. Continuous curves show the total heat capacity in both cells.

We found that the experimental data for the coefficient $\sigma$ deviate significantly from the high temperature calibration lines for both cells, as shown in Fig. 3 This deviation can be explained by the additional heat capacity due to surface Majorana QPs (Eq. 3). We converted these experimental data into the corresponding specific heats using Eq. (7). In Fig. 4 shows the obtained temperature dependence of the heat capacity, normalized to the heat capacity of Bogolyubov quasiparticles. As in Fig. 3 black squares correspond to experimental data of cell *A*, and blue triangles correspond to data of cell *B*. We fitted this data with Eq. (4) with *Z* as the fitting parameter and found that $Z = 0.94 \pm 0.02$. The excellent results of this fit are shown in Fig. 4 by solid lines. This unexpectedly shows that the Majorana specific heat corresponds to a smooth surface of the cell walls (see Eq. 3), despite the fact that the surface of the copper foils looks rough. Another unexpected result is the value of the Majorana heat capacity, which corresponds to a surface of the order of magnitude larger than the geometric one. This contradiction was resolved after examining the surface with an electron microscope.

## The surface of the copper walls after annealing

**Figure 5:**
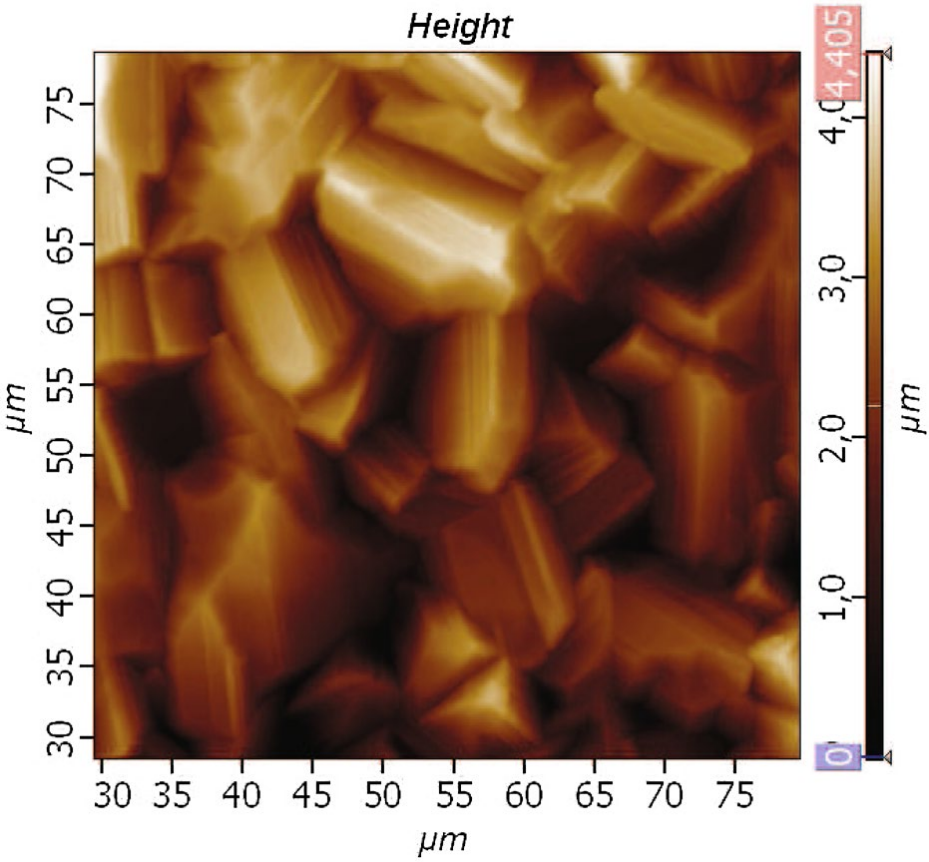
The electron microscope photo of cooper surface after an annealing in oxygen. The few layers of monocrystals enlarge the atomically smooth surface on a few times.

All parts of a nuclear demagnetization refrigerator are usually annealed in a rarefied oxygen gas. This process removes free hydrogen, the ortho-para conversion of which results in multi-day heat generation. We examined the surface of copper foils after annealing with an electron microscope. Photograph of the surface shown in Fig. 5. We found that the entire surface of foils is covered with several layers of copper single crystals with a size of about 10 microns. This size is 100 times greater than the coherence length, and therefore, for Majorana particles, the surface of single crystals is an ideal atomically smooth surface. It is seen that the effective copper surface is several times larger than the geometric one and depends on the thickness of the single crystal layer. Therefore, an increase in the effective surface by an order of magnitude is quite possible. Measurements of gas adsorption on the copper surface showed its increase by an order of magnitude, but the accuracy of these studies is not great.

This result sheds light on other experiments in the ultra low temperature range. In particular, the formation of a deep layer of single crystals of copper apparently explains the difference in the results of the experiments in Grenoble and Lancaster, where a paper cell was used^13^. In particular, in the Grenobles experiment on studying the second-order quenching transition in quantum media^22^, it was found that the emerging quantum vortices live long enough to measure their total energy. However, when scattered over a smooth surface, they should have collapsed in a very short time. It is very possible that the vortices were fixed on the protrusions of the single crystals and therefore lived long enough.

## Impact of solid $^3$He

The question that could be stated now if there are any other possible sources of additional heat capacity besides the Majorana QPs. The fact that we measured the surface states and determined the strong influence of *S*/*V* ratio leads to the assumption about possible influence of the ${^3}$He layers adsorbed on the walls of the cell. Indeed, in our experiments this influence was strongly suppressed because the surface had been covered by ${^4}$He during the condensation of the liquid ${^3}$He into the cell. One can suggest that there are some islands of adsorbed ${^3}$He remained in the cell which could give the additional heat capacity to the bulk ${^3}$He. But there are experimental facts that show that it is not the case. The precise measurements of the heat capacity of adsorbed ${^3}$He in the interesting for current experiments temperature range were done in^24^. It was found out that the heat capacity of adsorbed ${^3}$He has the magnetic nature. In this case the magnetic heat capacity of ${^3}$He nuclei should decreases with increasing temperature as $C_{magn}\sim T^{-2}$^30^.

**Figure 6:**
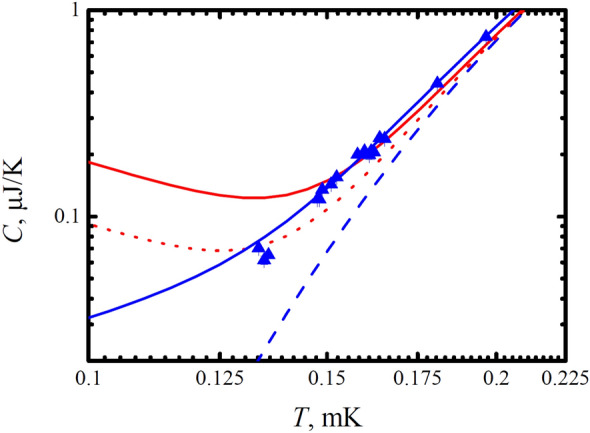
Dependence of the heat capacity of superfluid ${^3}$He on the temperature in the cell *B*. The dashed blue line shows the calibrated heat capacity based on results obtained above 0.25 $T_C$. The solid blue line shows the dependence of the heat capacity with the addition of Majorana quasiparticles. The red dotted and solid lines show possible dependences in which the Majorana heat capacity is replaced by the hypothetical heat capacity of solid ${^3}$He as $AT^{-2}$. It is clearly seen that the $AT^{-2}$ dependence cannot adequately describe the experimental data.

In order to check this assumption, we tried to describe the experimental points by the sum of the heat capacity of the Bogolyubov quasiparticles and the $AT^{-2}$ dependence using the quantity *A* as an adjustable parameter. Fig. 6 shows two fitting curves. On one of them, the *A* value was chosen in accordance with the results obtained at a temperature of 0.15 $T_C$, and on the other, with the results at 0.13 $T_C$. It is clearly seen that the curves with the $AT^{2}$ dependence cannot adequately describe the experimental data. On the contrary, a fit using the heat capacity of Majorana quasiparticles fits the experimental data perfectly.

Another reason which allows us to exclude the impact of adsorbed ${^3}$He is that the temperature decay after the typical heating pulse in our experiments is well described by single exponential function while the presence of the adsorbed ${^3}$He leads to the two exponential behaviour of the decay after heating event^31^ due to the imperfect thermal contact between the solid and liquid ${^3}$He.

## Conclusion

Thus, we can conclude that the existence of Majorana quasiparticles has been experimentally confirmed in superfluid ${^3}$He-B by direct measurements of its heat capacity in cells with different surface-to-volume ratios.
